# A Primary Study of Variable Polarity Plasma Arc Welding Using a Pulsed Plasma Gas

**DOI:** 10.3390/ma12101666

**Published:** 2019-05-22

**Authors:** Zhenyang Lu, Wang Zhang, Fan Jiang, Shujun Chen, Zhaoyang Yan

**Affiliations:** 1 Engineering Research Center of Advanced Manufacturing Technology for Automotive Components, Ministry of Education, Beijing University of Technology, Beijing 100124, China; lzy@bjut.edu.cn (Z.L.); wang_zhang0731@163.com (W.Z.); sjchen@bjut.edu.cn (S.C.); zhygyan@126.com (Z.Y.); 2 Beijing Engineering Researching Center of laser Technology, Beijing University of Technology, Beijing 100124, China; 3 Chemical and Materials Engineering, University of Alberta, Edmonton, AB T6G 2R3, Canada

**Keywords:** variable polarity plasma arc welding (VPPAW), weld formation, pulsed plasma gas, arc voltage

## Abstract

A process variant of variable polarity plasma arc welding (VPPAW), that is, the pulsed plasma gas VPPAW process, was developed. The pulsed plasma gas was transmitted into the variable polarity plasma arc through a high-frequency solenoid valve to modify the output of the plasma arc. The collection of arc electrical characteristics, arc shapes, and weld formation from VPPAW, double-pulsed VPPAW (DP-VPPAW), and pulsed plasma gas VPPAW (PPG-VPPAW) was carried out to examine if the pulsed plasma gas was able to play a positive role in improving the stability and quality of the VPPAW process. The arc voltage shows that the pulsed plasma gas had a greater influence on the electrode positive polarity voltage. The lower the plasma gas frequency was, the lower the arc voltage fluctuation frequency was and the greater the arc voltage fluctuation amplitude was. From the arc image, it could be observed that the arc core length had a short decrease during the general rising trend after plasma gas was turned on. The arc core width only had a slight change due to the restriction of the torch orifice. Compared with pulsed current wave, the pulsed plasma gas could better enhance the fluidity of the molten pool to reduce porosity during aluminum keyhole welding.

## 1. Introduction

Variable polarity plasma arc welding (VPPAW) has been widely used in aeronautics, astronautics, and the automobile industry to produce high-quality and high-precision weld joints of aluminum alloy [1,2]. The constrained process in the plasma arc torch leads to high energy density and arc stiffness of the plasma arc, but makes the output of the arc coupled. This characteristic results in a smaller weld lobe curve than for other processes, and leads the keyhole molten pool easily affected by environmental changes in complex welding environments [3]. All of these cause a poor dynamic stability of the keyhole and weld defects, which restrict the application of VPPAW.

Increasing arc energy density is a common method to improve welding quality. The laser hybrid plasma arc welding process [4,5] can improve the energy density and stability of the plasma arc by benefiting from interactions with laser beams. The gas-focusing plasma arc welding process [6], with arc column secondly constricted by focusing gas, can improve the arc restraint degree and stability significantly. The increase in arc stability effectively reduces the disturbance from welding arc to molten pool, but it is difficult to eliminate the other influence on molten pool stability. 

Increasing the robustness of the keyhole molten pool is more helpful for the interference resisting. The controlled pulsed plasma arc welding process [7,8], which makes the holes in periodic opening and closing state by adjusting the current output, could effectively improve the robustness of the molten pool. The double-pulsed VPPAW (DP-VPPAW) [9,10] adds additional high-frequency pulsed current into electrode negative (EN) and electrode positive (EP), which makes the molten pool oscillate periodically to improve stability of the welding process and quality. However, the additional current parameters lead the thermal-force synchronous change and cause difficulties of process control. 

The vibration-assisted plasma arc welding process [11,12], which uses mechanical vibration to drive molten pool vibration, is an efficient method to decrease the attendant heat fluctuation of the plasma arc, compared with the current wave. These methods produced satisfactory process effectiveness, but the mechanical coupling of vibration system and welding torch reduced the stability and precision of the plasma welding torch structure.

The previous studies [13,14] have shown that the plasma gas flow rate significantly affects the pressure output, but for the heat output, it is insignificant. Based on this, a novel welding process named pulsed plasma gas VPPAW (PPG-VPPAW) was proposed in this study. A specially designed plasma arc torch was used to control plasma gas flow, and the pulsed plasma gas VPPAW system was developed. This paper focuses on the arc behavior and welding process with PPG-VPPAW. The reason for the periodic variation of arc voltage and arc profile during PPG-VPPAW is discussed based on the arc electric signals and arc image acquired from experimental results. Furthermore, the weld-forming experiments were carried out to explore the reason for the improving the fluidity of the molten pool and reducing porosity during aluminum keyhole PPG-VPPAW.

## 2. Experimental Procedure

### 2.1. Experimental System

**Figure 1:**
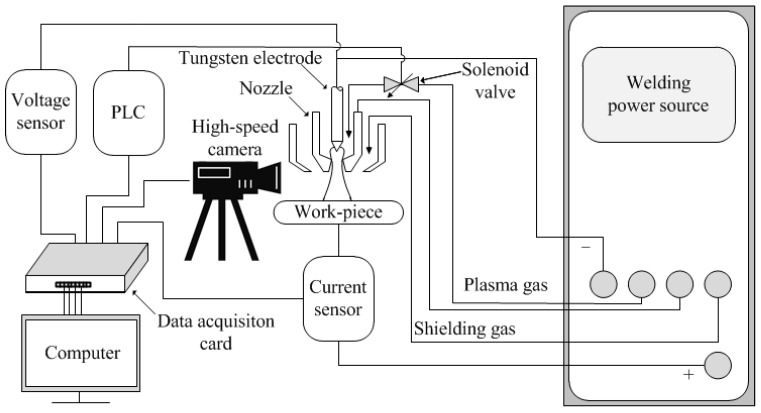
Schematic diagram of the PPG-VPPAW system.

Figure 1 shows the schematic diagram of the PPG-VPPAW system, which included three parts: the pulsed plasma gas control unit, the VPPAW system, and the data acquisition system. The plasma gas control unit consisted of a S7-200 series PLC and a 35A series high-frequency solenoid valve (rated voltage of DC24V, rated power of 5.4 W, conduction response time of 6 ms, outages response time of 2 ms, and the maximum atmospheric flux of 16.2 L/min for argon). The solenoid valve was installed in the plasma gas tube of the plasma arc torch and as close to the torch nozzle as possible to generate the pulsed plasma gas flow. The VPPAW system consisted of a welding power source, a modified plasma arc torch, and the needed accessories. The data acquisition system mainly consisted of a voltage sensor, a current sensor, a high-speed camera, a data acquisition card, and an industrial computer. During the welding process, the welding current and voltage were collected by the current and voltage sensors. The plasma arc was imaged by the high-speed camera (IDT Y4 series), which was set to focus on the fixed region around the nozzle of the unmovable plasma arc torch. The experimental data was displayed and recorded by the data acquisition card in the industrial control computer.

### 2.2. Experimental Design

**Figure 2:**
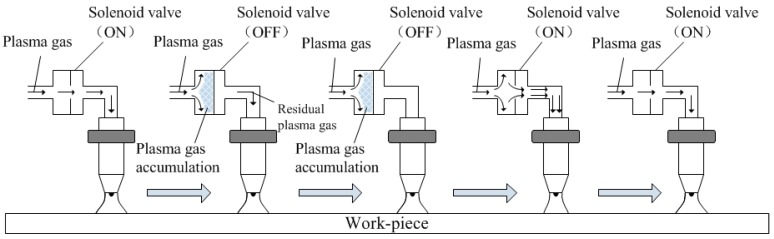
The principle of PPG-VPPAW process.

The principle of the PPG-VPPAW process is shown in Figure 2. When the solenoid valve was closed, the plasma gas flow was blocked by the solenoid valve and gathered at the entrance of the solenoid valve. When the solenoid valve was opened, the blocked plasma gas flow was released and input into the plasma arc at a velocity greater than the set value at that moment. Then, the plasma gas flow returned to the set value and waited the next closure of the solenoid valve.

**Figure 3:**
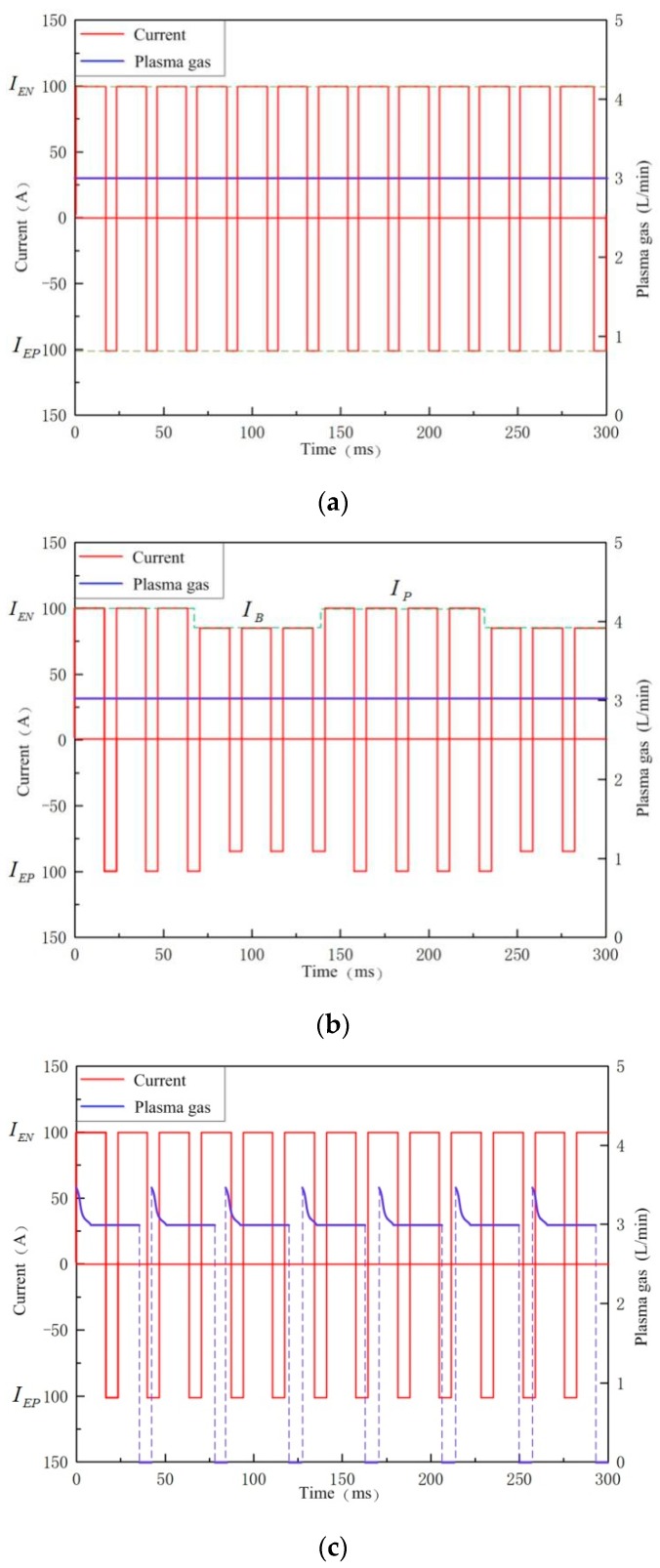
Schematic diagram of current–plasma gas flow waveform. (**a**) VPPAW; (**b**) DP-VPPAW; (**c**) PPG-VPPAW.

In order to study the effect of pulsed plasma gas acting on the plasma arc, the arc electric signals and arc image were acquired from PPG-VPPAW and compared with those in VPPAW and DP-VPPAW. The current and plasma gas flow waveforms of the VPPAW, DP-VPPAW, and PPG-VPPAW processes are shown in Figure 3. The VPPAW process has the variable polarity square current wave with unequal straight and reverse polarity time intervals, and the plasma gas flow rate remains constant, as shown in Figure 3a. For the DP-VPPAW process, the current wave has been decreased periodically, the total current wave presents two periodically varying pulses (i.e., a high-frequency variable polarity pulse and a low-frequency pulse), and the plasma gas flow rate is the same as for the VPPAW process, as shown in Figure 3b. In the PPG-VPPAW process, the current waveform is the same as for the VPPAW process while the plasma gas flow rate is input intermittently in the form of a pulse, as shown in Figure 3c. Based on a large number of trials, the process parameters in this study were selected in Table 1 to make the comparison clear. In Table 1, $I_{EN}$ was the electrode negative current; $I_{EP}$ was the electrode positive current; $I_{B}$ was the basic current in DP-VPPAW; $I_{P}$ was the pick current in DP-VPPAW. The arc image capture rate was 3000 fps and the signal sampling rate was 10,000 Hz. The plasma gas and the shielding gas both were pure argon.

In order to study the effect of pulsed plasma gas on the molten pool in the keyhole welding process, the weld-forming experiments were carried out. The weld bead geometries and porosity distribution from the VPPAW, DP-VPPAW, and PPG-VPPAW processes were investigated. In order to study the influence of the pulsed plasma gas on the molten pool, the filling wire was not applied for avoiding the effect of filling material on molten pool behavior and simplifying the experimental model. In these experiments, 5 mm thick 5A06 aluminum alloy was selected as the work piece. The plasma gas and the shielding gas both were pure argon. Based on a large number of trials, the parameters in this part were selected to obtain a good weld bead geometry, as shown in Table 2.

## 3. Results and Discussion

### 3.1. Variation in Arc Electrical Signal

**Figure 4:**
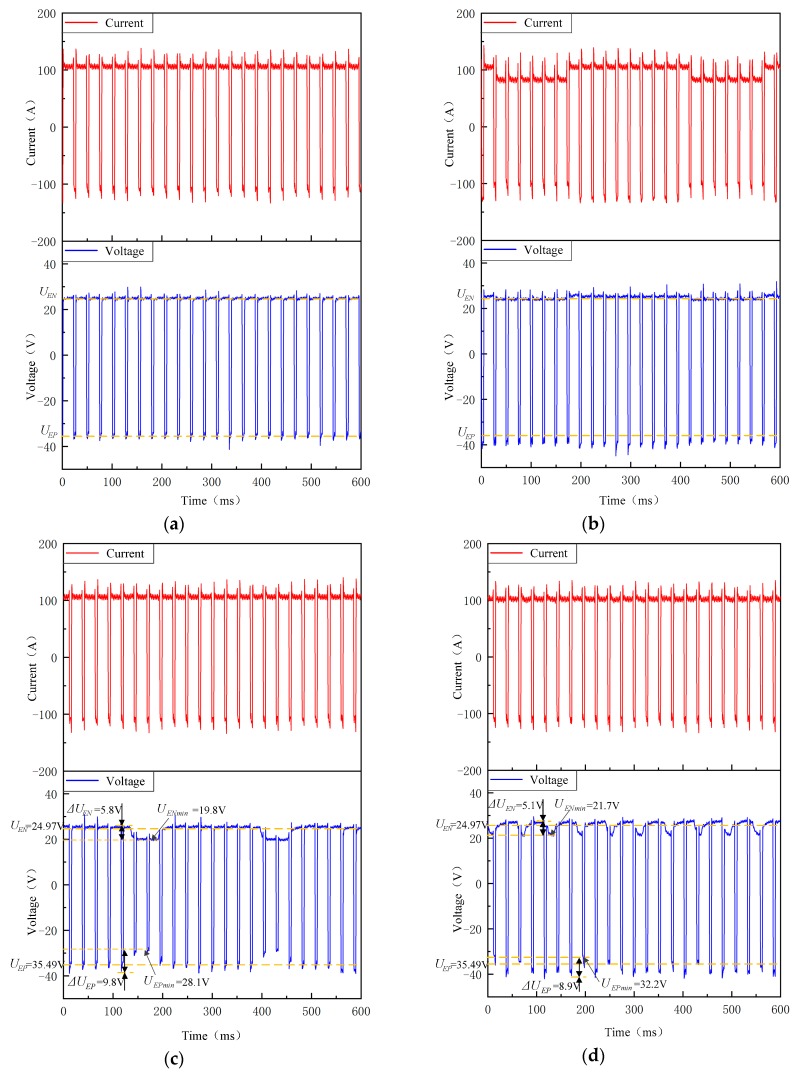
Welding current–voltage synchronizing wave form. (**a**) VPPAW; (**b**) DP-VPPAW; (**c**) PPG-VPPAW (4 Hz); (**d**) PPG-VPPAW (20 Hz).

The welding electrical signals of VPPAW (Experiment 1-1) and DP-VPPAW (Experiment 1-2) are shown in Figure 4a,b, respectively. It can be observed that the current waves well fit the preset parameters. The root mean square (RMS) and absolute mean (AM) values of current are shown in Table 3. In comparison with traditional VPPAW, the current of PPG-VPPAW was almost unchanged. Figure 4c,d show the welding electrical signals of PPG-VPPAW under different plasma gas flow pulse frequencies with the same plasma flow rate; the plasma gas flow pulse frequencies were 4 Hz (Experiment 1-3) and 20 Hz (Experiment 1-4), respectively. Compared with Figure 4a,c, it can be observed that the current wave had a negligible effect on the pulsed plasma gas, while the arc voltage fluctuated periodically with the pulsed plasma gas frequency. The electrode negative period voltage ($U_{EN}$) in the VPPAW process and solenoid valve on state of the PPG-VPPAW process was 24.97 V on average, and the electrode positive period voltage ($U_{EP}$) was 35.49 V on average. When the solenoid valve was off state and the gas flow pulse frequency was 4 Hz, the $U_{EN}$ and $U_{EP}$ decreased by 5.8 V and 9.8 V, respectively. When the gas flow pulse frequency was 20 Hz, the $U_{EN}$ and $U_{EP}$ decreases were 5.1 V and 8.9 V, respectively. The above results showed that the lower the frequency of plasma gas, the more distinct the influence on the arc voltage waveform. At a certain plasma gas pulse frequency, the pulsed plasma gas has a greater influence on the electrode positive polarity voltage.

When the plasma gas injects into the arc column, it requires more energy to keep enough ionized particles that let the current through. When the plasma gas is shut off, the required energy which was used to ionize the gas decreases, and the arc voltage also decreases. This decrease relates to the shut-off time of plasma gas and has a maximum value. With the frequency of plasma gas increased, the shut-off time decreases with lack of time to let the arc voltage decrease to the maximum value. In the EP phase of the PPG-VPPAW process, due to the cathodic cleaning phenomenon [15], the size of the arc profile is larger than that in the EN phase, which lets it be more affected by the plasma gas shut-off. 

**Figure 5:**
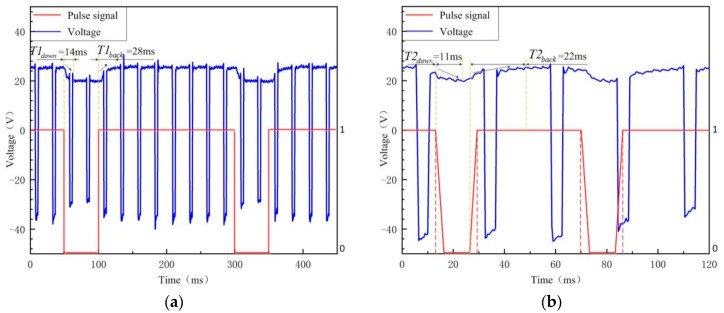
The arc voltage of PPG-VPPAW. (**a**) PPG-VPPAW (4 Hz); (**b**) PPG-VPPAW (20 Hz).

Figure 5 displays more details about the arc voltage of PPG-VPPAW with the plasma gas flow pulse frequencies of 4 Hz and 20 Hz. The pulse signals of “1” and “0” indicate that the solenoid valve is in the on state and off state, respectively. It can clearly be seen that with the plasma gas flow pulse frequency of 4 Hz, the arc voltage is dropped immediately to the minimum voltage when the plasma gas is shut off, which costs 14 ms, and the voltage decrease rate is 0.44 V/ms. When the plasma gas is turned on, it takes 28 ms to return the average voltage, and the voltage increase rate is 0.20 V/ms accordingly. Once the plasma gas flow pulse frequency increases to 20 Hz, the voltage decrease rate is nearly same as that in 4 Hz (0.46 V/ms), but it is hard to find a stable minimum voltage due to the lack of shut-off time of the plasma gas. When the plasma gas is turned on in 20 Hz situation, the voltage recovery time is 22 ms, and the voltage increase rate is 0.23 V/ms accordingly. The above results show that the plasma arc needs more time to recover the effect of plasma gas closure, and the plasma gas flow pulse frequency has less effect on the voltage decrease rate.

When the plasma gas is shut off, there is no plasma gas injecting immediately and the arc zone could be considered as a relative closed environment and establish balance easily. Compared with it, when the plasma gas is turned on, the plasma gas continuously injects into the arc column and makes it harder to establish balance, so the arc voltage needs more time to stabilize after the plasma gas is turned on. Furthermore, the higher the plasma gas frequency is, the lower the plasma gas accumulation rate is, so the arc voltage needs less time to stabilize after the plasma gas is turned on. The voltage decrease rate is correlated with arc characteristic, but there is no significant correlation with the frequency of plasma gas. However, when the frequency of plasma gas is above a certain value, the plasma gas is turned on again when the arc voltage is not yet decreased to the stable voltage without plasma gas, so the recovery speed of the voltage becomes faster.

### 3.2. Variation in Arc Profile

**Figure 6:**
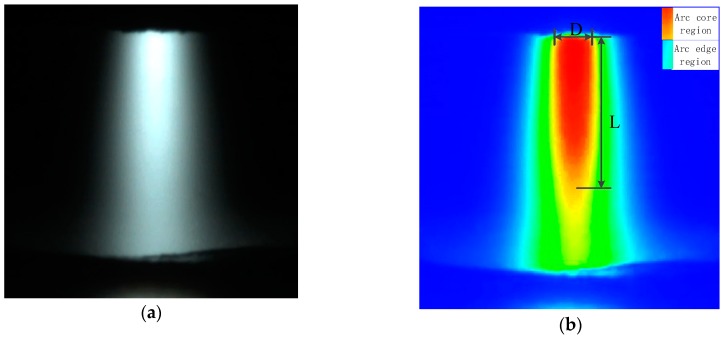
Edge extraction and regionalization of welding arc. (**a**) Original arc image; (**b**) processed arc image.

In order to better observe and analyze the variation in arc profile collected by the high-speed video camera, it is necessary to divide the arc region for regionalization and measurement. The arc image processing procedure is shown in Figure 6. The original arc image is shown in Figure 6a. Figure 6b is the colored gray-scale image that is transformed from the original arc. According to the intensity of the arc light, the arc can be divided into 256 scale levels. Yang et al. illustrated that the gray scale was larger with higher intensity in the arc gray-scale image, with 90% of the arc intensity as the arc core region [16,17]. The gray-scale image was colored by RGB for the convenience of arc core region [18,19], the red and green region of processed arc image were the core and edge region, respectively. As a result, the arc profile would be measured and analyzed using the arc core region, as presented in Figure 6b. The arc profile, which was defined by arc core length L and arc core width under the nozzle D, was accurately measured by computerized measurement technique.

**Figure 7:**
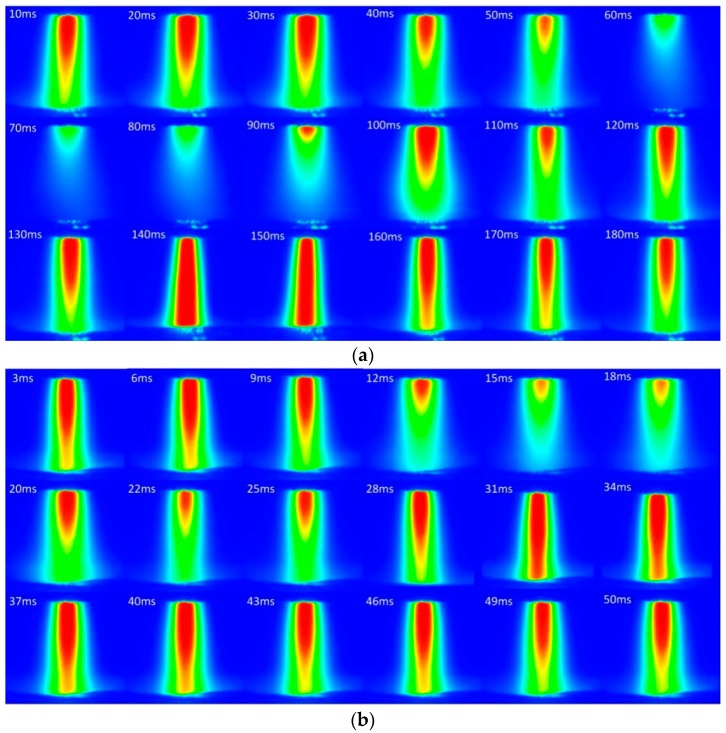
Comparison of arc images at negative polarity stage. (**a**) PPG-VPPAW (4 Hz); (**b**) PPG-VPPAW (20 Hz). (The color bar of this figure is same as Figure 6).

**Figure 8:**
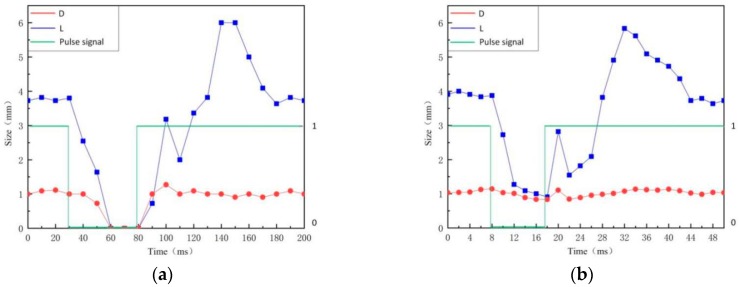
Arc core region size of PPG-VPPAW. (**a**) PPG-VPPAW (4 Hz); (**b**) PPG-VPPAW (20 Hz).

Figure 7a displays the variation of arc profile in a complete plasma gas cycle of PPG-VPPAW with the 4 Hz plasma gas pulse frequency in EN phase. Figure 8a shows the corresponding L and D, which were measured using the method mentioned above. From 0 to 30 ms, the arc images were relatively stable, the L and D were about 3.76 mm and 1.05 mm, respectively. When the plasma gas was shut off, from 30 ms to 60 ms, the arc constricted rapidly with the L and D decreased to 0 mm. From 60 ms to 80 ms, the core region of the arc almost disappeared because there was no plasma gas supply. When the plasma gas was turned on, from 80 ms to 100 ms, the core region of the arc significantly increased with L and D increased to 3.18 mm and 1.27 mm, respectively. Then, the L had a decrease to 2.07 mm at 110 ms and increased again to 6 mm in 140 ms. After that, the L gradually decreased to 4.09 mm from 140 ms to 170 ms and then maintained in a stable state. In this period, the D only had a slight change due to the restriction of the torch orifice. The above results show that the arc core length had a short decrease during the general rising trend after plasma gas was turned on. These two peaks of arc core length were due to the initial overshoot phenomenon. Once the plasma gas is turned on, the overshoot velocity is higher than the setting value, which causes the subsequent plasma gas to not keep up, leading the L to decrease. 

Figure 7b shows the variation of arc profile in a complete plasma gas cycle of PPG-VPPAW with the 20 Hz plasma gas pulse frequency in the EN phase. The corresponding L and D are shown in Figure 8b. It can be seen that the change of PPG-VPPAW arc profile with 4 Hz and 20 Hz plasma gas pulse frequencies have the same tendency. As mentioned before, due to the increase of plasma gas pulse frequency, there is not enough shut-off time of plasma gas to affect the arc profile fully, the L cannot decrease to 0 mm from 8 ms to 18 ms, and the first peak value is also less than that in 4 Hz. In the whole cycle with 20 Hz plasma gas pulse frequency, the core region of the arc always exists, and the D keeps balanced at 1.02 ± 0.17 mm.

**Figure 9:**
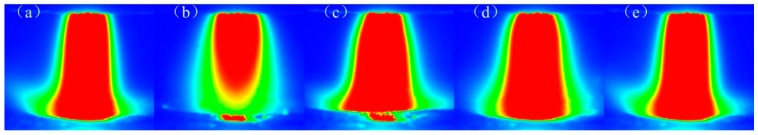
Comparison of arc images at positive polarity stage. (The color bar of this figure is same as Figure 6).

Figure 9 displays the arc profile of EP phase in different VPPAW processes and stages. Figure 9a shows the arc profile in the VPPAW process. Figure 9b,c show shutting-off and turning-on stages, respectively, with 4Hz PPG-VPPAW. Figure 9d,e show shutting-off and turning-on stages, respectively, with 20 Hz PPG-VPPAW. Compared with these arc profiles, it is easy to find that only in the shut-off stage with 4 Hz PPG-VPPAW process, the arc core length L significantly decreased. This phenomenon cannot be found when the plasma gas pulse frequency increased to 20 Hz. That means the influence of plasma gas shutting off in the EP phase of the VPPAW process is much smaller than in the EN phase, especially in a high plasma gas pulse frequency. This is because the arc core region is directly related to the temperature field, which has such a great inertia that the temperature change could not quickly respond to the disturbance. When the plasma gas frequency increases, the effect of pulsed plasma gas on the EP phase is harder to observe. 

### 3.3. Variation in Welding Formation

**Figure 10:**
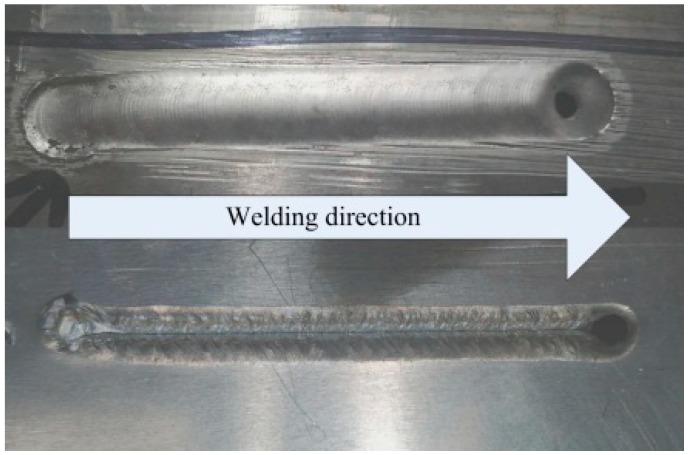
The front and back of the PPG-VPPA (20 Hz) weld joint.

**Figure 11:**
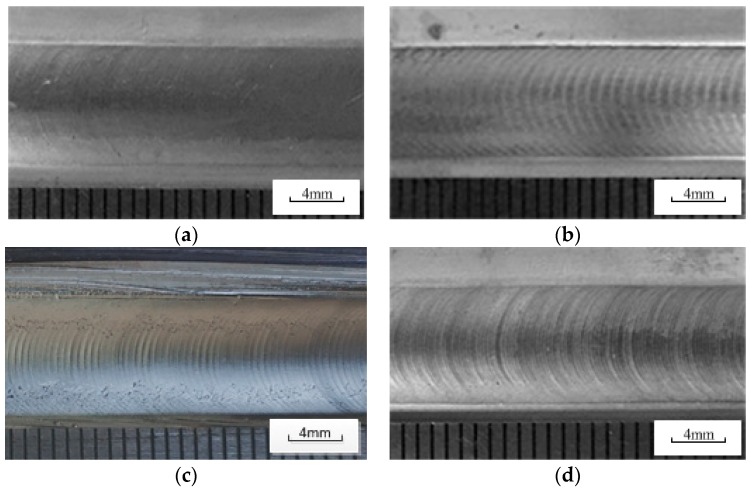
Morphology of weld surface. (**a**) VPPAW; (**b**) DP-VPPAW; (**c**) PPG-VPPAW (20 Hz); (**d**) PPG-VPPAW (40 Hz).

Figure 10 shows the front and back of the PPG-VPPA (20 Hz) weld joint. It can be clearly seen that the keyhole is completely penetrated at the end of the weld joint. The weld joint formed as shown in Figure 10 is due to the lack of metal filling and the molten pool flow toward the front of the weld. Figure 11 shows the appearance of the weld surface with different welding methods. A smooth weld joint profile of VPPAW is shown in Figure 11a. As shown in Figure 11b–d, both DP-VPPAW and PPG-VPPAW form a fish-scale pattern on the surface of the weld joint due to the stirring action on the welding pool caused by the periodical oscillation of the arc pressure. The ripples formed on the weld joint surface show that the fluidity of the welding pool has been enhanced, and benefits the welding quality. Compared with Figure 11b,c, it is clearly demonstrated that the plasma gas frequency has a stronger effect acting on the welding formation than current frequency under the premise of acceptable welding formation. Compared with Figure 11c,d, the denseness of ripple profile on the weld joint surface is directly proportional to the frequency of the plasma gas.

**Figure 12:**
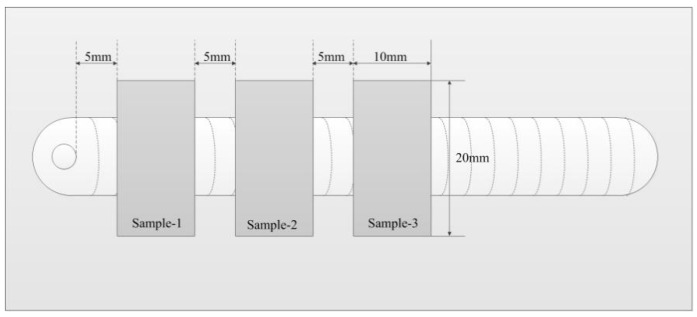
The preparation method of the sample.

**Figure 13:**
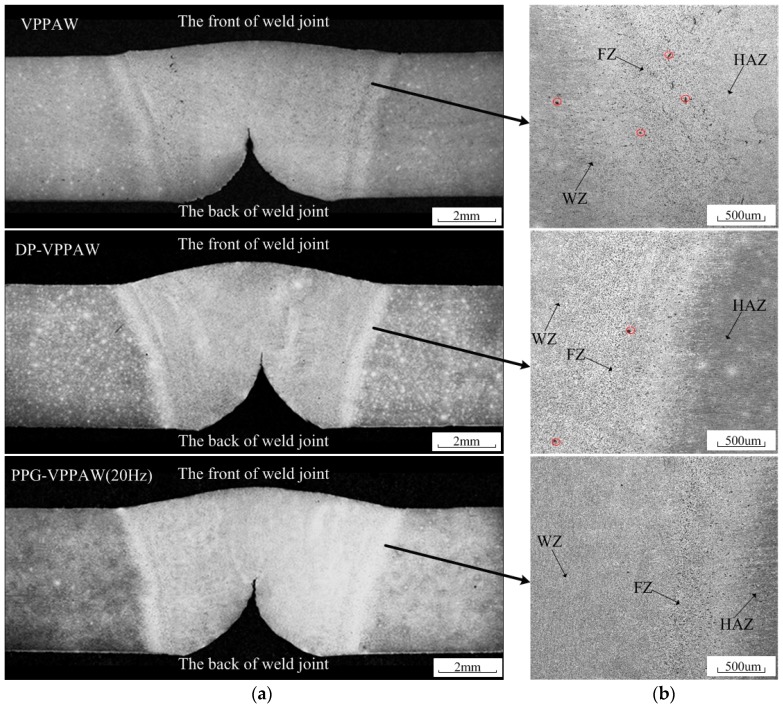
Macrographs of cross-section and the distribution of pores in weld fusion line region. (**a**) Macrographs of cross-section; (**b**) the distribution of pores in weld fusion line region.

The preparation method of the sample is shown in Figure 12. Three samples were selected in every weld joint. We observed that the characteristics of the three samples of the same weld joint were basically the same. Therefore, a sample of each weld joint was randomly selected for further analysis. The cross-sections of the weld joint with different welding methods are exhibited in Figure 13a. The average weld reinforcement and width are measured as shown in Table 4. The VPPAW process and PPG-VPPAW process have nearly the same current wave, so the weld width from these two processes are similar. For the DP-VPPAW process, due to the decrease of current in the low frequency, the weld width also decreases. The distribution of porosity in the weld fusion line region with different welding methods is shown in Figure 13b, where WZ is weld zone, FZ is fusion zone, and HAZ is heat-affected zone. It shows clearly that the porosity has appeared both in fusion zone and weld zone from the VPPAW process, and the porosity could be observed in the fusion zone from the DP-VPPAW process. In contrast, there is no observable porosity in the cross-section from the PPG-VPPAW process. The results of arc electrical characteristics and arc profile show that PPG-VPPA periodically fluctuates due to the periodic variation of plasma gas flow rate. The molten pool oscillates periodically under the oscillating arc. Therefore, the pulsed plasma gas has a stronger effect than the pulsed current wave which enhances the fluidity of the molten pool and the spillover probability of the porosity from the molten pool increases [20,21].

## 4. Conclusions

This study proposed a novel plasma arc welding process, named pulsed plasma gas variable polarity plasma arc welding process, and investigated the effect of pulsed plasma gas on the arc electrical signal, arc profile, and welding formation. The results can be summarized as follows:

(1) The shut-off time is the key factor affecting arc behavior under the pulsed plasma gas, and this effect is stronger in the EP phase of the variable polarity plasma arc process.
(2) In the EN phase, the length of arc core region is also affected by the shut-off time of plasma gas. Due to the overshoot, two peak values of arc core length appear in the return of plasma gas. The pulsed plasma gas has less effect on arc profile in the EP phase.
(3) Compared with pulsed current wave, the pulsed plasma gas could better enhance the fluidity of the molten pool to reduce porosity during aluminum keyhole welding.

## Figures and Tables

**Table 1:** Parameters for arc image and electrical signal comparison.

**Number**	**Arc Length (mm)**	**Tungsten Setback (mm)**	**Shielding Gas Flow Rate (L/min)**	**Plasma Gas Flow Rate (L/min)**	$I_{EN}:I_{EP}$ **(A)**
1-1	6	4	10	3.0	100:100
1-2	6	4	10	3.0	100:100
1-3	6	4	10	3.0	100:100
1-4	6	4	10	3.0	100:100
**Number**	$I_{B}:I_{P}$ **(A)**	**Electric Pulse Frequency (Hz)**	**Electric Pulse Duty Cycle**	**Solenoid Valve Frequency (Hz)**	**Solenoid Valve Duty Cycle**
1-1	-	-	-	-	-
1-2	80%	3	5:3	-	-
1-3	-	-	-	4	4:1
1-4	-	-	-	20	4:1

**Table 2:** Parameters for weld-forming experiments.

**Number**	**Arc Length (mm)**		**Tungsten Setback (mm)**	**Shielding Gas Flow Rate (L/min)**	**Plasma Gas Flow Rate (L/min)**	$I_{EN}:I_{EP}$ **(A)**
2-1	5		4	10	3.0	130:150
2-2	5		4	10	3.0	130:150
2-3	5		4	10	3.0	130:150
2-4	5		4	10	3.0	130:150
**Number**	$I_{B}:I_{P}$ **(A)**	**Welding Speed (mm/s)**	**Electric Pulse Frequency (Hz)**	**Electric Pulse Duty Cycle**	**Solenoid Valve Frequency (Hz)**	**Solenoid Valve Duty Cycle**
2-1	-	1.83	-	-	-	-
2-2	80%	1.83	3	5:3	-	-
2-3	-	1.83	-	-	20	4:1
2-4	-	1.83	-	-	40	4:1

**Table 3:** The root mean square (RMS) and absolute mean (AM) values of current.

Welding Methods	RMS (A)	AM (A)
VPPAW	100.95	100.66
DP-VPPAW	93.56	92.39
PPG-VPPAW(4 Hz)	100.98	100.66
PPG-VPPAW(20 Hz)	100.84	100.08

**Table 4:** The weld reinforcement and width.

Welding Methods	Weld Width (mm)	Weld Reinforcement (mm)
VPPAW	10.18	0.64
DP-VPPAW	9.64	0.82
PPG-VPPAW	10.01	0.73
